# Comics as a Physical Education Tool for Health Promotion in Brazilian Primary Education, Based on Paulo Freire’s Principles of Empowerment

**DOI:** 10.3390/children10091575

**Published:** 2023-09-19

**Authors:** George Bernard Soares Nascimento, Marcelo de Maio Nascimento, Luciana Márcia Gomes de Araújo, Élvio R. Gouveia, Andreas Ihle

**Affiliations:** 1 Department of Physical Education, Federal University of Vale do São Francisco, Petrolina 56304-917, Brazil; georggn@hotmail.com (G.B.S.N.); luciana.marcia95@gmail.com (L.M.G.d.A.); 2 Department of Physical Education and Sport, University of Madeira, 9020-105 Funchal, Portugal; erubiog@uma.pt; 3 Laboratory of Robotics and Engineering Systems (LARSYS), Interactive Technologies Institute, 9020-105 Funchal, Portugal; 4 Center for the Interdisciplinary Study of Gerontology and Vulnerability, University of Geneva, 1227 Carouge, Switzerland; andreas.ihle@unige.ch; 5 Department of Psychology, University of Geneva, 1227 Carouge, Switzerland; 6 Swiss Center of Expertise in Life Course Research LIVES, 1227 Carouge, Switzerland

**Keywords:** Paulo Freire, knowledge strategies, posture education, comics, childhood education

## Abstract

Incorrect postural habits developed already at an early age are predictors of low back pain and functional limitations in adult life. Postural education programs (PEPs) are activities developed in Physical Education classes with the aim of promoting healthy habits. One tool used by PEPs is comics. The objective of this study was to develop comics and apply them as a teaching tool in PEPs for students aged seven to ten years. The procedures were based on individual empowerment principles, including creation activities, reading, painting, crosswords, and discussion of comics. The activities strengthened the students’ interactions, gaining new knowledge that required cognitive and expressive resources to interpret, associate, and conceptualize themes of correct body posture. During six weeks of intervention, knowledge about body posture, anatomy, and health promotion exercises increased significantly in relation to the beginning of activities. The comic book proved to be an effective, attractive, and low-cost didactic resource.

## 1. Introduction

Among the challenges that Brazilian school teachers face in their day-to-day practices is the search for teaching methods and tools that are innovative, motivating, and effective. Due to a series of colonial historical and cultural issues, associated with the modus operandi of political conduct over the years, Brazilians have to face a series of economic and social inequalities, which directly affect the area of education [1]. In view of this, there are many barriers that educators encounter in order to achieve an effectively transformative pedagogical practice, capable of contributing to the formation of critical and reflective citizens who can contribute to the construction of their communities and country in the future [2].

Teaching is not limited to classes—rather, it is part of a bilateral relationship between student and teacher in relation to the topics that will be addressed, and the choice of motivating tools [3]. In general, there will be no teaching if there are not enough mechanisms to promote learning [4]. Learning takes place, among others, based on a series of experiences and knowledge formed by the exchange of information between student and teacher, and not simply by reproducing content, which characterizes a mechanical and uncritical process [5]. An alternative to meet the pedagogical needs of teachers and improve the teaching–learning process consists of incorporating didactic materials that include the language of young students. This strategy can increase student engagement in activities, enhancing learning [6].

The use of attractive topics, languages or teaching tools by the student both facilitates learning and is capable of empowering the student. Empowerment occurs when those involved discover their socio-transformative power through educational actions [7]. In Brazil, the term empowerment became popular in the 1980s with the work *Fear and Boldness: The Daily Life of the Teacher*, written by the pedagogue and philosopher Paulo Freire [8]. Freire’s studies were intended to raise the education levels of Brazilians from the lower social classes. According to Freire, through education, low-income individuals and communities would free themselves from the socio-political and cultural oppression exercised for decades by the dominant classes. In Freire’s theory, empowerment was not intended to form new individuals, but to make those involved more critical and innovative [9].

From this perspective, the gain in knowledge is presented as a foundation for young people to transform decision-making in their daily lives, including the development of new life habits in relation to health, quality of life and well-being [10]. Thus, the sooner young people receive knowledge in the area of health mediated by simple and engaging didactic resources, the greater the chances of not developing risky behaviors for their future health [11]. It is worth mentioning that for the processes to achieve their objectives, the type of strategy chosen for the transfer of knowledge is crucial for effective learning. Therefore, when the contents are motivating and are understood by the students, the greater the chances that the set of lived experiences will be added to the previous knowledge. Thus, in the future, this new knowledge can be multiplied and used in problem solving [12]. Concomitantly, this process reflects what we previously called empowerment [2,8].

Among the many educational tools used in schools in recent decades to enhance teaching and learning, there are comics, which feature a simple, engaging and fun material [13]. Around the world, comics are considered basic elements of popular culture [14], used as a teaching tool in schools [15]. In a review study that investigated the use of comics in schools compared to traditional texts [16], it was found that teachers and students preferred comics due to the playful and relaxed way in which the stories addressed complex themes of the day by day. However, there was no consensus whether texts or comics would offer better learning for students.

In Brazil, where this study was carried out, the first comics were created in 1934 in the Globo newspaper, in special inserts for children [17]. Later, in 1937 and 1939, the initiative was expanded to the inserts “Globo Juvenil e Infantil” and in the insert “Gibi”, respectively. For this reason, even today, the word “Gibi” is used in Brazil as a synonym for comics. Over the years, comics have been part of Brazilian childhood and adolescence, winning over many admirers. Thus, in 1988 [18], comics were incorporated by the National Curricular Parameters as an official methodology for teaching and learning in schools. An important step towards legitimizing comics as a teaching tool took place in 2006, when the National School Libraries Program included comics in the Ministry of Education’s book shopping list [19,20]. Since then, researchers have highlighted the advantages of using comics as an educational resource with high potential to integrate curricular components and strengthen knowledge in schools [21,22].

Considering the Brazilian socioeconomic context, the use of comics in schools is interesting because this tool, in addition to being easily accessible for low-income families, can be adapted to the language of students and their parents. Moreover, regardless of age group or level of prior knowledge [23], comics are simple to handle and attractive [24]. During the reading, the characters strengthen the imaginary universe of the readers, involving themselves through the retraction of particularities that suggest identification with the approached situations.

### Comics, Physical Education and Health Promotion

The relationship between comics and the discipline of Physical Education was established in the 1970s, when the government of the military dictatorship used comics through a character called “Dedinho” to encourage young people to practice team sports in schools [25]. Since then, interest in using comics in Physical Education classes has grown, mainly because of the possibility of approaching different themes through stories [26]. In Brazil, comics even proved to be effective during the period of the coronavirus pandemic, when they were associated with online teaching as an alternative to approach different themes, including the body–movement relationship, in a fun and colorful way [27]. A previous study carried out in Brazil highlighted the use of comics as a playful tool to strengthen basketball teaching [28]. Another application of comics in Physical Education classes was their integration in postural education programs [29].

At school, the Physical Education discipline seeks to promote physical and mental health through the practice of physical exercises, sports, and leisure activities. Among the different contents covered, there are postural problems that result from incorrect habits, especially when students remain seated in the classroom for a long time [30], as well as the use of very heavy backpacks [31]. Postural problems in schoolchildren are even worse when the young person has sedentary habits [32]. All of this can contribute to the onset of pain and health problems in adulthood. The prevalence of back pain increases with age: at 7 years the incidence is 1%, increasing to 6% at 10 years, reaching 12% at 12 years, and 18% among 14- to 16-year-olds [33]. Back pain has a multifactorial origin, as it is associated with occupational problems (i.e., recurrent incorrect posture).

Over the years, epidemiological studies have reported the causes and consequences of back pain in children and adolescents [34,35]. Incorrect postural habits developed at an early age are predictors of low back pain and functional limitations in adult life [36]. For this reason, postural problems are part of the list of diseases considered a global public health issue [37]. A strategy for the prevention and treatment of postural problems and back pain in the young population is the creation of postural education programs (PEPs), which consist of a set of educational actions that disseminate knowledge on how to adopt and maintain correct body posture [38,39]. In a systematic review study, Noll, Candotti and Vieira [40] analyzed PEPs developed in Brazilian schools to prevent back pain in children aged 6 to 14 years. According to the authors, the studies showed that the activities were effective, and consisted of the association between theoretical and practical classes. Topics addressed included the anatomy of the spine, basic principles of kinesiology, teaching the correct way to sit, pick up objects from the ground or from high places, and the ideal weight and correct use of backpacks, in addition to the importance of regular practice of physical exercises to strengthen the musculoskeletal structures. Moreover, among the didactic tools used in Brazilian Physical Education classes, one was comics [29].

Although comics are used in Physical Education classes to strengthen teaching and learning practices, there are still gaps in the literature about their impact on students’ education, as well as the best way to use them. A current review that included 56 studies on the use of comics in schools revealed that most investigations focused only on reading the texts [16]. The authors also concluded that until then, topics such as the construction of stories by students and the impact of comics on learning had not yet been exhaustively worked on. Regarding the Brazilian context, it is worth noting that a review study showed that PEPs with or without the use of comics were developed primarily with the population residing in the South and Southeast regions [40], where economic and social development is higher than in the Northeast region of Brazil [41]. Therefore, studies carried out with school students residing in the Northeast region, especially in cities far from large urban centers, can contribute to the creation and improvement of health and education surveillance strategies, benefiting territorial development. Thus, this study aimed (1) to create comic books, (2) to use them as a teaching tool in a postural education program for students aged seven to ten, and (3) to verify the impact of comics on the knowledge and postural attitudes of young students.

## 2. Materials and Methods

### 2.1. Study Design and Participants

This is an educational intervention study with a quantitative and qualitative approach in accordance with the desired objectives [42]. In this aspect, the research used a mixed method to collect data and present results. This approach proved to be ideal for obtaining data through contact and interaction with young students and teachers (i.e., experiences), as well as for the development of the PEP (i.e., comics and postural education). The sample was non-probabilistic and intentional. The set of PEP activities included 56 students of both sexes (9.4 ± 0.8 years), students from two classes at a municipal school in the city of Petrolina, located in the Northeast region of Brazil. All students from both classes agreed to participate in the study, in addition to their teachers. Inclusion criteria were: (1) being enrolled in school and (2) having at least 75% presence in the PEP. Exclusion criteria were: (1) those whose parents did not sign the informed consent form to participate in the study, (2) students who did not sign the special informed consent form for children, and (3) students who did not complete all stages of the study. The activities of the present study were associated with a project that evaluated the levels of physical activity of schoolchildren. The actions were approved by the Ethics and Research Committee of the University of Vale do São Francisco, under protocol nº 2769034.

### 2.2. Postural Education Program

The set of PEP activities developed were based on principles of Physical Education that sought to empower students according to health principles [2,11]. Therefore, the use of comics sought to stimulate interaction between students, strengthening their potential. Through learning about postural habits, students were empowered in different ways, including the exercise of autonomy, responsibility, respect, empathy, as well as the understanding of their rights as citizens to receive education/information about health care. To ensure that students from both classes received the same information during the PEP, the 56 students carried out the activities together (Phase V). Only in the retention of knowledge period (Phase VI) did the two classes perform physical exercises and sports separately. The activities took place between March and July 2019, and were developed in seven phases:

Phase I (literature review): Preceding all activities, a theoretical study was carried out to support the other phases of the investigation.

Phase II (visiting the school): At this moment, the first contact with students and teachers was made. During the visit to the school, there was a conversation with the students to assess their knowledge about the anatomy of the spine, correct body posture, and the ideal weight and the correct way to carry the backpack. On this day, both the students and their backpacks were weighed so that the correct weight of the backpack could be calculated. Knowing that the weight of the backpack could vary between days of the week, due to additional materials (i.e., books and sports equipment), the control took place on the day of the Physical Education class, as it was the day on which the students carried the heaviest load.

Phase III (creation of comics): The activities focused on creating stories and strategies for using comics in the classroom. The planning of activities was essential because it considered the child’s ability to negotiate the reading and interpretation of images and stories. The way the child interprets the comics is processed by a system of codes that sometimes works independently, and is sometimes able to interact with the facts [20].

Phase IV (pilot study): This activity aimed to test the comics tool with schoolchildren. For that, a group of 22 students were invited to participate in a class with comics. Subsequently, these students were not part of the sample of the main study. After the pilot, there was an evaluation of the material and method of application. Afterwards, a few adjustments to the methodological procedures were performed.

Phase V (execution of the PEP): The activities took place for six weeks (one class per week, 50 min). Each class had 5 moments: (1) the initial debate with a presentation of the theme of the day. In this, we sought to facilitate children’s understanding of each new content; (2) individual reading of the comic; (3) reading and discussion of comics in groups of 5 to 8 students; (4) activities in the comics: painting parts of the human body, crosswords, finding seven mistakes in the images; and (5) correction of group activities, followed by discussion and closure of the class. During the meetings, students were encouraged to reflect on their postural attitudes at school and at home, as well as being asked to compare their postural habits with the teachings of comic figures.

Phase VI (retention of knowledge): To assess the effectiveness of learning about body posture through comics, the students remained for four weeks without receiving any type of knowledge related to postural habits. During this period, they only participated in activities consisting of physical exercises and sports games.

Phase VII (second assessment of knowledge): At this stage, the effectiveness of learning was tested through a new comic book, containing the following activities: (1) identifying in the images and marking the correct posture on how to correctly lift an object from the ground, how to pick up an object from a shelf above the head, and how to carry a heavy object; (2) identifying and marking on the images the correct way to carry a backpack, as well as the most suitable type of backpack; (3) drawing parts of the human body, detailing the spine, specifying the lumbar, thoracic and cervical regions; (4) identifying in the images and marking the correct way to sit on a chair at school and at home; and (5) creating a story, making drawings and writing dialogues between characters considering body posture. To verify the retained knowledge, the number of correct answers for tasks 1 to 4 was quantified, and the results are shown in the following section. At that moment, the second weighing of the backpacks was also carried out to verify possible changes in the students’ habits. Another strategy used was to ask the two classroom teachers whether the students’ postural habits had changed since the beginning of the PEP (in the last 5–6 weeks).

## 3. Results

The study included 27 boys (9.3 ± 0.9 years old) and 29 girls (9.5 ± 0.7 years old). Table 1 presents the set of activities developed, considering the phases of planning, execution and evaluation of the knowledge gained by the students through the postural education program.

The first phase of activities (literature review) was developed by the executing team at the university, on the premises of the Physical Education course/UNIVASF. Through the second phase, it was possible to collect important information about the prior knowledge of the 56 students. Thus, there was a lack of underlying knowledge related to the topic of postural education. So, based on the information gained, we moved on to the third phase. The pilot (Phase III) was developed at the school with 25 students of the same age group as those who participated in the PEP. However, these were excluded from the study. Through the pilot, it was found that the students’ level of knowledge on topics related to the human body (i.e., anatomy of the spine), body posture, weight and correct type of backpack was extremely low. Through the pilot, it became clear to the executing team that the comics tool should present motivating and simple-to-understand elements. Therefore, we began to prepare materials that focused on the following points: (1) we created characters and stories close to the social context of the students; (2) to facilitate the fixation of information mediated by the comics, each of the characters received a name; (3) the comic characters’ clothes were created with cheerful colors; and (4) the comic book also received a section of coloring activities, another with questions and answers, and a third section with a game to identify seven mistakes and crosswords.

**Figure 1:**
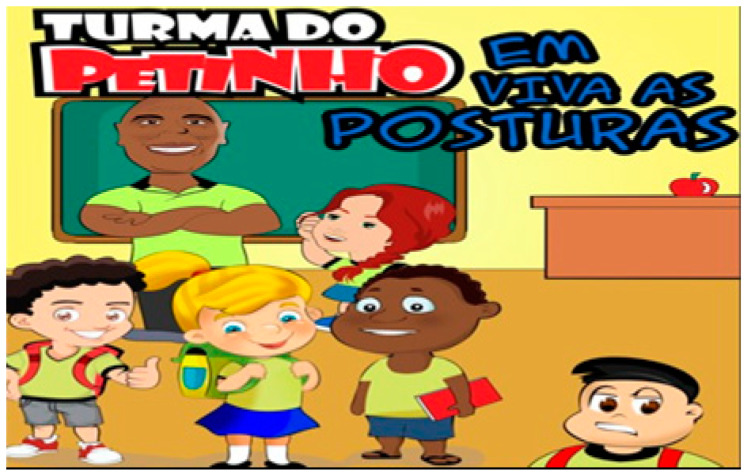
Comics cover image: “Team Petinho and body postures”.

Figure 1 shows the main cover of the created comics. A strategy used to motivate the reading of the material was to portray some members of the postural education program team.

**Figure 2:**
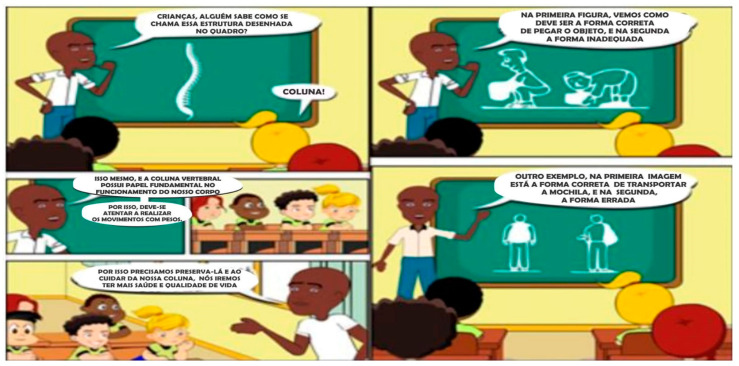
Figures on the left side (from top to bottom): In the first scene, the teacher asks the students if anyone knows the name of the structure presented. One of the student answers: spine. In the second scene below: the teacher confirms the student’s answer and highlights the role of the spine in carrying out daily tasks. In the third scene: the teacher emphasizes taking care of the spine to maintain good health and quality of life. Figures on the right side (from top to bottom): In the first scene, the teacher shows comparatively the correct and wrong way to lift an object from the ground. In the second scene, the correct and wrong way to carry a school backpack is presented.

Figure 2 presents the main character of the PEP story, entitled “Chico”. This character was a young man with inadequate and recurrent postural habits. The other characters in the comics were his classmates and his school’s PE teacher. The dialogues between the characters were composed of lines that sought to teach Chico the importance of changing his postural habits. The creation of imaginary scenarios sought to reproduce the school environment, as well as the students’ homes. This strategy was important because, during the activities, students were encouraged to reflect on their postural attitudes at school and at home.

**Figure 3:**
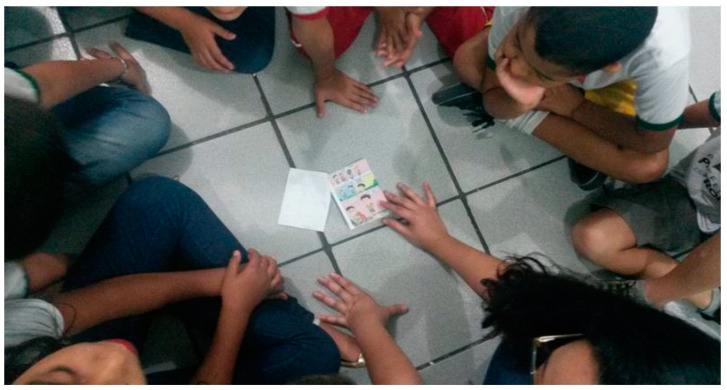
Reading activities and discussing the stories, followed by filling in the characters’ speeches.

Figure 3 illustrates one of the moments in which, after creating and writing dialogues between characters drawn in their comic books, the students were instructed to read and reflect on the topic of body posture. These activities encouraged young students to interact and exchange knowledge, as well as suggest strategies for health care in the classroom.

**Figure 4:**
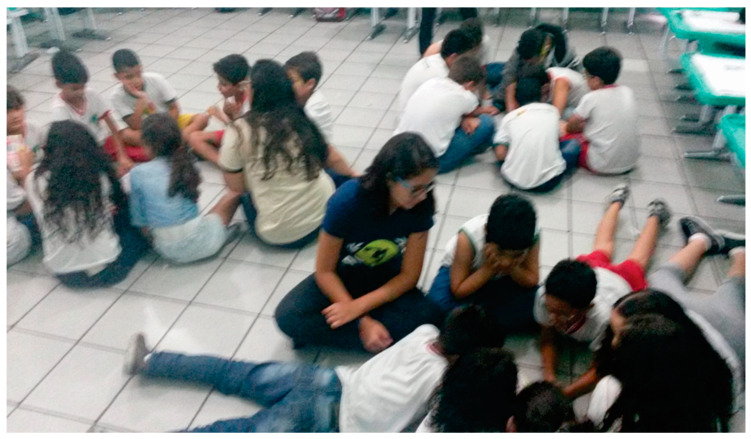
Group comic reading activities, examples of images on spine themes, correct posture when sitting, and correct carrying of the backpack.

Figure 4 shows moments of the postural education program in which students interacted, discussing topics related to correct body posture, strengthening the possibility that comics offer for learning in a playful and interactive way.

After six weeks of the PEP intervention, the group participated in four weeks of Physical Education classes that included physical exercise and sports (volleyball and football). During this period, students did not receive any information about body posture. At the end of these four weeks, the students were reassessed regarding their body posture. Table 2 shows students’ performance on reassessment tasks after the intervention program. It is noteworthy that right at the beginning, before the program activities (week 1), all 56 students did not have any knowledge about the PEP theme. However, after the intervention period (weeks 19–20), the group achieved a knowledge rate between 94 and 100% in the four learning categories covered by the program (Table 2).

Regarding the weight of the backpack and how to carry it, it was found that at the beginning of the study, before reading the comics (week 2), 60% of students carried backpacks with loads greater than 10% of their body mass, and only 40% carried backpacks with an adequate weight (≤10% of body mass). Before the intervention, 17% of the students had backpacks with two straps to carry school supplies, which is ideal, while 83% used equipment with a single strap, favoring postural problems. At the end of the study (week 20), 93% of students had backpacks with adequate weight (≤10% of their body mass). Furthermore, the number of students who wore backpacks with two straps (correct distribution of the load between two shoulders), which was 10 young people at the beginning of the study, increased to 52 individuals at the end of the study. This represented a 75% improvement regarding the type of backpack used.

After the intervention with comics (week 20), the teachers responsible for the two groups of students reported having observed positive postural changes in the young people’s behavior during classes. The statements were made based on the knowledge they had received in the students’ lectures. It is noteworthy that the two teachers participated in the activities voluntarily and willingly, as until then, they were unaware of the topics covered by the PEP. Another gain provided by PEP to students was knowledge about anatomy (i.e., conceptualization of segments of the human body) and ergonomics.

## 4. Discussion

Our study aimed to create comic books, use them as a teaching tool in a postural education program, and verify the impacts of the activities on the knowledge and attitudes of a group of students aged seven to ten years. The results suggested that after four weeks of intervention, students were trained in the basic fundamentals of spinal anatomy, understood the importance of transforming their postural habits in the classroom to promote spinal health, and a significant number adopted backpacks with two straps and reduced the weight of the material taken to school. Furthermore, it was found that during the implementation of the PEP and in the following four weeks (period of knowledge retention), the students maintained correct body habits and carried the correct weight in their backpacks. Therefore, considering that before the implementation of the PEP, students were unaware of all the topics covered, the use of comics in Physical Education classes proved to be an effective strategy to promote health and well-being among young students. Our findings were in line with a previous study also carried out in Brazil, which highlighted comics as a practical, well-accepted and effective tool for improving students’ knowledge, interaction and autonomy in the classroom [21]. In turn, we also corroborate an investigation carried out with Brazilian schoolchildren (7–11 years old), which associated comics in Physical Education classes with a focus on postural education [29].

The literature highlights that the use of comics in the classroom favors the reflection of new information and consequently the gain of knowledge in a complementary way to the traditional teaching standards adopted in schools [43]. The findings were in line with previous studies that highlighted the value of comics in sensitizing people of different age groups about diseases and the need to promote health [44,45]. Comics are a teaching tool that can both stimulate students’ creativity and strengthen interactions between them. Through the association between comics and Physical Education, students experienced a way of learning about body and health that valued creativity and expression [5]. An interesting outcome of this study was exploring the potential already highlighted in comics [20]. The practice of reading comics and analyzing images proved to be a strategy capable of strengthening the potential of Physical Education classes to disseminate information to promote the health, well-being and quality of life of young students [46].

The adopted strategy of portraying in comics the members of the team responsible for the PEP, including the school faculty, benefited students’ motivation and adherence to activities. At the end of each class, the comics were taken by the students to their homes. In this way, knowledge gained in the classroom was shared with family and friends. This strategy allowed information about correct body posture to be disseminated throughout the community [47]. In general, our results corroborated the activities of PEPs previously carried out with Brazilian students [29,48].

Thus, based on Paulo Freire’s vision of a socio-transformative perspective that can be achieved through educational measures, the inclusion of comics in Physical Education classes proved to be a transformative pedagogical tool [2,8] which motivated students to gain new knowledge and transform habits, improving knowledge that can benefit one’s health and well-being.

The present study has some limitations. First, its observational design does not allow for establishing causal mechanisms, as well as attributing the results to other samples. Secondly, as this is a study based on the creative and expressive abilities of those involved, it is possible that emotional and environmental factors may have generated biases influencing the results. In general, the students looked forward to the day and time of the PEP activities with high expectations. However, motivational issues arising from playfulness are part of Physical Education classes [49]. Third, it was not possible to present lasting changes in classroom postural habits and the weight and type of backpack lasting longer than a period of six weeks (Phase V–VI) after the end of the PEP implementation (Phase IV). Therefore, it is suggested that future studies adopt a longitudinal follow-up of more than three months to verify the retention of PEP gains among students. Another suggestion is to include a control group to compare two different methodologies based on comics. Among the strengths of the present study, we can highlight the gains regarding the impact of the PEP based on the comic book tool on young Brazilian students at a public school located in a peripheral area—in particular, the detailing of didactic procedures for each phase of the work. Thus, documentation of actions can help future Physical Education teachers in creating new postural education projects.

## 5. Conclusions

Our results showed that through comics it was possible to promote the knowledge of 56 students aged seven to ten years about health education in Physical Education classes. By reading comics and engaging in painting activities, crosswords, and looking for seven postural errors in pictures, students understood and internalized the content of the PEP. Thus, knowledge in the areas of anatomy and kinesiology was incorporated. The findings confirmed previous studies that suggested comics as a teaching tool with high potential to be adopted in the classroom. It is expected that the information presented in this study can support actions in the areas of health education, as well as encourage Physical Education professionals to incorporate comics into their daily practices.

## Figures and Tables

**Table 1:** Set of activities developed by the postural education program.

Phase/Week	Activity	Contents
I	Literature review	Postural education programs, Physical Education for schoolchildren, postural deviations common in schoolchildren, exercises to prevent postural deviations, and use of comics in classes of Physical Education
II	1st Evaluation Visiting the school	
1	Previous knowledge: (a) Correct body posture; (b) Spinal column; (c) Weight/transport of the backpack.	Talk to students about topics related to spinal anatomy, body posture, and backpack
2	Habits	Backpack weighing
III	Creation activities	
3	Comics	Creation of stories and figures with a focus on postural education
4	Comics	Creation of texts and scenarios in the Corel draw program.
5	Comics	Creation of texts and scenarios in the Corel draw program.
6	Comics	Preparation of strategies for reading comics by students, considering the association between entertainment and the transmission of health concepts
IV	Pilot Testing the effectiveness of teaching tools	Application/testing of comics with 22 students. * Participants were excluded from the study.
8	Examination of materials and methods	Assessment of students’ degree of understanding of the proposed procedures, Modification of materials and methods.
V	Intervention	
9	1st class	(a) Reading the comics (story: anatomy), (b) Painting of images about the human body, parts of the spine.
10	2nd class	(a) Reading the comics (story: postural deviations and back pain), (b) Find seven errors in the pictures of the comics
11	3rd class	(a) Reading the comics (story: correct and incorrect body position to sit at school and at home), (b) Design and color the classroom chairs and tables.
12	4th class	(a) Reading the comics (story: correct and incorrect position of the body to lift objects off the floor, pick up objects on shelves, and carry heavy objects), (b) Design and color the classroom chairs and tables
13	5th class	(a) Reading the comics (history: correct and incorrect position to carry the backpack, ideal backpack weight), (b) Tell a story to the colleague.
14	6th class	(a) Reading the comics (story: the importance of physical exercise for health, and the prevention of postural problems), (b) Drawing in comics: people doing physical exercise.
VI	Knowledge retention	
15–18	Physical Education practical classes	Practice of physical exercises and games
		* The activities did not address the postural education theme.
VII	2nd Evaluation	
19	Learning through comic	Application of new comics containing questions, crosswords, and activities for drawing and coloring
20	Habits	Second weighing of backpacks and questioning the teachers.

Note: * = Participants were excluded from the study.

**Table 2:** Students’ performance in assessing retained knowledge about correct body posture after the intervention program.

Image on Comics	Yes [*n* (%)]	No [*n* (%)]
Posture and Objects		
Recognized the correct way to pick up objects on the floor.	55/56 (98%)	1/56 (2%)
Recognized the correct way to pick up objects from above.	53/56 (94%)	3/56 (6%)
Recognized the correct way to carry heavy objects.	49/56 (88%)	10/56 (12%)
Schoolbag		
Recognized the correct way to carry the backpack	56/56 (100%)	-----
Recognized the best types of backpacks to transport school supplies.	54/56 (96%)	2/56 (4%)
Anatomy		
Draw and identify parts of the spine.	56/56 (100%)	-----
Identify postural deviations in the images.	56/56 (100%)	-----
Posture when sitting		
Correct posture when sitting in the classroom chair.	56/56 (100%)	-----
Correct posture when sitting in front of the computer.	56/56 (100%)	-----

Note: “-----“ = No results.

## Data Availability

The data presented in this study are available upon request from the corresponding author.
